# Experimental manipulation of monoamine levels alters personality in crickets

**DOI:** 10.1038/s41598-018-34519-z

**Published:** 2018-11-01

**Authors:** Robin N. Abbey-Lee, Emily J. Uhrig, Laura Garnham, Kristoffer Lundgren, Sarah Child, Hanne Løvlie

**Affiliations:** 1 Dept of Physics, Chemistry and Biology, IFM Biology, Linköping University, 58183 Linköping, Sweden; 2 Department of Biology, Southern Oregon University, 1250 Siskiyou Blvd, Ashland, OR 97520 USA; 3 Faculty of Biology, Medicine, and Health, Manchester University, Michael Smith Building, Dover St, Manchester, M13 9 UK

## Abstract

Animal personality has been described in a range of species with ecological and evolutionary consequences. Factors shaping and maintaining variation in personality are not fully understood, but monoaminergic systems are consistently linked to personality variation. We experimentally explored how personality was influenced by alterations in two key monoamine systems: dopamine and serotonin. This was done using ropinirole and fluoxetine, two common human pharmaceuticals. Using the Mediterranean field cricket (*Gryllus bimaculatus*), we focused on the personality traits activity, exploration, and aggression, with confirmed repeatability in our study. Dopamine manipulations explained little variation in the personality traits investigated, while serotonin manipulation reduced both activity and aggression. Due to limited previous research, we created a dose-response curve for ropinirole, ranging from concentrations measured in surface waters to human therapeutic doses. No ropinirole dose level strongly influenced cricket personality, suggesting our results did not come from a dose mismatch. Our results indicate that the serotonergic system explains more variation in personality than manipulations of the dopaminergic system. Additionally, they suggest that monoamine systems differ across taxa, and confirm the importance of the mode of action of pharmaceuticals in determining their effects on behaviour.

## Introduction

Animal personality (i.e., consistent among-individual variation in behaviour), has been described in a broad range of species^1,2^. Despite research demonstrating that animal personality can have important ecological and evolutionary consequences^2–4^, the factors shaping and maintaining variation in personality are still poorly understood. Underlying genetic variation has been demonstrated in a number of species, but our understanding of the mechanisms translating genetic variation into personality variation is generally limited^5,6^. This calls for rigorous experimental studies using experimental manipulations of different mechanistic pathways in order to understand how genetic variation is translated into personality variation^7–9^.

Aspects of personality have been linked to monoaminergic systems^2,10,11^, including variation in metabolite levels, methylation, and gene polymorphisms for both dopamine and serotonin. Dopamine levels, polymorphisms and differential methylation of dopamine-associated genes are related to novelty-seeking and exploratory behaviour in mammals and birds^2,7,12–14^. Dopamine is also involved in the recovery of aggression after social defeat in insects^15,16^. Additionally, serotonin levels are negatively associated with aggressiveness in several species^2,10,17^, but positively related to activity and aggression in others^13,14,18^. Polymorphisms in serotonin transporter genes are related to aggression, anxiety, and impulsivity^2^. Such evidence suggests monoamines may be one of the mechanisms translating genetic variation into personality variation^7,8^. However, we cannot yet clearly describe the link between monoamines and behaviour, and further work exploring the causality of observed relationships between neuroendocrinology and personality is needed.

We experimentally manipulated two key monoamine systems to determine their effect on personality. For our manipulations we used human pharmaceuticals: ropinirole, which alters the dopaminergic system, and fluoxetine, which alters the serotonergic system. Ropinirole is a dopamine receptor agonist that has been linked to motor control and is prescribed to treat Parkinson’s disease and restless legs syndrome^19,20^. Fluoxetine is a selective serotonin reuptake inhibitor prescribed to treat depression and anxiety^21^. We used pharmaceuticals for our manipulations because they have known effects on human personality and behaviour, and as monoamine systems are evolutionarily conserved across taxa^22^, these compounds are good candidates for potentially explaining personality variation in other species. We used the Mediterranean field cricket because they are a model species for neuroethological studies^23–25^, have been shown to demonstrate personality^26^, and respond to monoamine manipulations^18,27,28^.

Based on previous work, we predicted that both of our monoamine manipulations would affect personality by increasing cricket activity^29,30^, and aggressiveness^15,18^, and that our serotonin manipulation would increase exploration tendency^13,14^. These three behaviours were chosen as they are consistent within individuals, describe personality types in a variety of species^31^, and are important to individual fitness^4^.

## Results

All raw data can be found as Supplementary Table S1. We confirmed that our behaviours were repeatable in our population by running repeatability analyses (for details see Methods below; Table 1), thus they can be classified as personality traits. This allowed us to assay individuals a single time for other parts of our study.

**Table 1:** Comparison of repeatability of cricket behavioural traits measured on 3 consecutive days (n = 24).

Behaviour	*R* (95% CI)
Activity	0.40 (0.14, 0.62)
Exploration	0.36 (0.06, 0.61)
Aggression	0.71 (0.27, 0.98)

Repeatability estimates (*R*) with 95% credible intervals (CI) are presented for the measured behaviours: Activity, distance moved in home environment (cm); Exploration, distance moved in novel area (cm); Aggression, winner of fight dyad, binomial.

**Figure 1:**
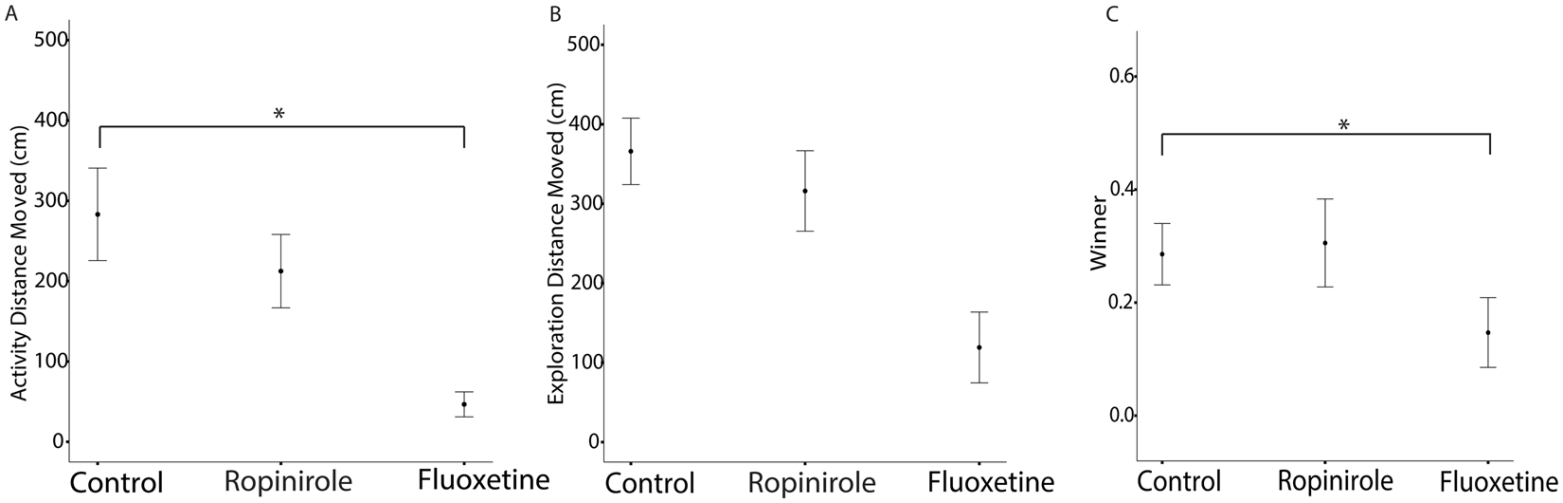
The influence of manipulation of monoamines (dopamine via ropinirole hydrochloride, or serotonin via fluoxetine hydrochloride) on cricket personality (n = 144). Mean and standard error of raw data describing (**A**) Activity, distance moved in home environment (cm); (**B**) Exploration, distance moved in novel area (cm); (**C**) Aggression, winner of fight dyad, binomial.

When comparing our dopamine-manipulated and unmanipulated individuals using linear models, there were no significant differences between groups in any of their behavioural responses measured (for details see Methods below; Table 2). Serotonin on the other hand did alter personality: our manipulated individuals had lower activity (distance moved in familiar environment) and lower aggression (more often lost fights) than control individuals (Table 2, Fig. 1). Exploration (statistically controlled for individual level activity), however, was not influenced by our serotonin manipulation.

**Table 2:** The influence of manipulation of monoamines (dopamine via ropinirole hydrochloride or serotonin via fluoxetine hydrochloride) on cricket personality (n = 144).

	Dopamine (ropinirole)	Serotonin (fluoxetine)
**A**. **Activity**		
Fixed Effects	β (95% CI)	β (95% CI)
Intercept^a^	9.35 (2.82, 13.18)	14.85 (10.52,19.13)
Monoamine Manipulation	2.41 (−1.42, 6.03)	**−10**.**32** (**−15**.**32**, **−4**.**94**)
Random Effects^b^	σ^2^ (95% CI)	σ^2^ (95% CI)
Injection Time	0.38 (0.19,0.61)	0.00 (0.00, 0.00)
Time Since Injection	0.00 (0.00, 0.00)	0.04 (0.01,0.12)
Colour marking	0.00 (0.00, 0.00)	0.03 (0.003,0.10)
**B**. **Exploration**		
Fixed Effects	β (95% CI)	β (95% CI)
Intercept^a^	276.6 (147.7, 396.6)	14.76 (9.12, 20.01)
Monoamine Manipulation	−26.79 (−155.9, 107.2)	−6.33 (−14.43, 2.24)
Activity	**0**.**23** (**0**.**03**, **0**.**43**)	0.00 (0.00, 0.00)
Random Effects^b^	σ^2^ (95% CI)	σ^2^ (95% CI)
Injection Time	0.00 (0.00, 0.00)	0.00 (0.00, 0.00)
Time Since Injection	0.00 (0.00, 0.00)	0.00 (0.00, 0.00)
Colour marking	0.06 (0.007, 0.24)	0.00 (0.00, 0.00)
**C**. **Aggression**		
Fixed Effects	β (95% CI)	β (95% CI)
Intercept^a^	−1.25 (−2.08, −0.46)	−0.61 (−1.28, 0.11)
Monoamine Manipulation	0.43 (−0.63, 1.51)	**−1**.**15** (**−2**.**34**, **−0**.**07**)
Random Effects^b^	σ^2^ (95% CI)	σ^2^ (95% CI)
Injection Time	0.00 (0.00, 0.00)	0.00 (0.00, 0.00)
Time Since Injection	0.00 (0.00, 0.00)	0.00 (0.00, 0.00)
Colour marking	0.00 (0.00, 0.00)	0.00 (0.00, 0.00)

Estimated effect sizes and 95% credible intervals (CI) around the mean of predictors of the measured behaviours: (A) Activity, distance moved in home environment (cm); (B) Exploration, distance moved in novel area (cm); (C) Aggression, winner of fight dyad, binomial. Significant differences (CI does not cross zero) are bolded.

^a^Reference category; control individuals.

^b^As proportion of total variance explained.

**Figure 2:**
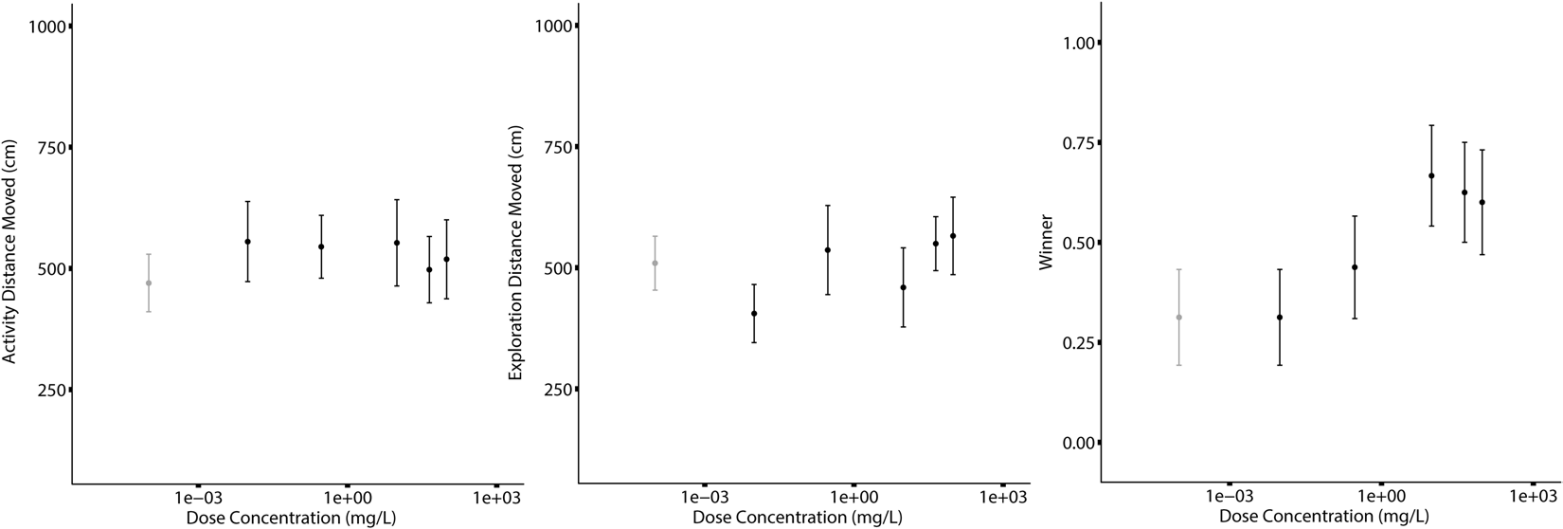
Dose response curve for cricket response to ropinirole injections. Mean and standard error of raw data describing (**A**) Activity, distance moved in home environment (cm); (**B**) Exploration, distance moved in novel area (cm); (**C**) Aggression, winner of fight dyad, binomial. Grey is the control group (concentration of zero) and black are the range of ropinirole doses.

We created a dose response curve across 6 concentrations of ropinirole and used linear models to confirm that only intermediately low (1 µM and 33 µM) doses tended to increase aggressiveness relative to control individuals (for details see Methods below; Table 3, Fig. 2). However, these results were only trends, confirming our observed lack of response to ropinirole in the main experiment.

**Table 3:** Comparison of different ropinirole concentrations on cricket personality (n = 96).

Fixed Effects	Activity	Exploration	Aggression
	β (95% CI)	β (95% CI)	β (95% CI)
Intercept (0 µM)	471.4 (326.2, 625.3)	438.4 (267.1, 606.3)	−0.79 (−1.88,0.25)
Activity Score	—	0.17 (−0.03, 0.36)	—
0.033 µM	82.03 (−130.0, 287.2)	−161.5 (−347.9, 37.2)	−0.22 (−1.75, 1.28)
1 µM	73.66 (−153.9, 276.4)	23.11 (−167.6, 232.1)	0.50 (−1.03, 2.01)
33 µM	81.76 (−138.1, 285.0)	−83.51 (−271.9, 110.6)	*1*.*49* (*−0*.*01*, *3*.*02*)
148 µM	25.15 (−182.9, 625.2)	35.56 (−146.9, 234.9)	*1*.*49* (*0*.*00*, *2*.*99*)
330 µM	46.84 (−161.0, 250.4)	44.40 (−124.5, 248.9)	1.20 (−0.32, 2.71)
**Random Effects**	**σ** ^**2**^ **(95% CI)**	**σ** ^**2**^ **(95% CI)**	**σ** ^**2**^ **(95% CI)**
Colour Marking	0.00 (0.00, 0.00)	0.00 (0.00, 0.00)	0.00 (0.00, 0.00)
Time of injection	0.10 (0.09, 0.12)	0.00 (0.00, 0.00)	0.00 (0.00, 0.00)
Time since injection	2915 (1882, 4525)	8926 (5154, 14340)	0.01 (0.01, 0.02)
Residual	88940 (60960, 114300)	64170 (46680, 87760)	1.00

Estimated effect sizes and 95% credible intervals (CI) around the mean of predictors of the measured behaviours: Activity, distance moved in home environment (cm); Exploration, distance moved in novel area (cm); Aggression, winner of fight dyad, binomial. Marginally significant trends are indicated with italics.

## Discussion

We show that our manipulations of serotonin causally affected the personality traits investigated by making crickets less active and less aggressive compared to unmanipulated crickets. Our manipulation of dopamine did not result in altered personality, despite testing a wide range of doses.

Our results add additional support confirming the often suggested link between monoamine systems and personality^2,10,11^. Specifically, our study adds further evidence of a causational relationship between serotonin manipulations and behavioural responses. Therefore, our study provides evidence that monoamines can be an underlying mechanism for personality variation.

Based on previous studies, we expected our manipulations of dopamine to also affect personality e.g.^13–15,30^. However, we found limited effects of our manipulations of the dopaminergic system by the use of ropinirole. Our dose-response experiment confirmed that the ropinirole concentration used tended to increase aggression in manipulated crickets, but we found no significant effects in any of our experiments. Our maximum dose was a high human therapeutic dose, but if there are significant differences in ropinirole sensitivity between insects and humans, potentially higher doses may be effective in insects and should be tested in future studies. Importantly, we used ropinirole which is highly selective for dopamine receptors, while other studies use less selective compounds (i.e. atypical antipsychotic medications like Fluphenazine that interacts with both dopaminergic and serotonergic systems, e.g. Rillich & Stevenson 2014) with different modes of action. The mode of action of specific compounds has been found to be important for the effect on animal species^32,33^. Many chemicals that alter the dopaminergic system also interact with the serotonergic system^34^, therefore, a testable explanation for the difference in results between our study and others could be the mode of action and specificity of the chemical used. Additionally, the specificity of ropinirole may influence its effectiveness across taxa and our results may indicate that dopamine receptors may differ in structure between at least humans and crickets.

Our results show that chemical manipulations of serotonin levels via fluoxetine injections changed individual behaviour and personality, adding further support to monoamines being key mechanisms in the maintenance of personality differences. Additionally, our findings support the larger body of work that indicates the complexity of monoamine systems and that their effect on behaviour can be dose, mode of action, and taxa dependent. Previous work and our results together highlight that the relationship between monoamines, behaviour, and personality may be highly dependent upon how the systems are manipulated. Thus, extensive future work is needed, focusing on categorizing behavioural responses to a large range of chemicals that alter monoamine systems in different, but specific ways (e.g., manipulations of only receptors vs. both receptors and transporters) as well as comparisons among monoamine systems (e.g. manipulations of single monoamine systems vs. multiple systems in conjunction) to better elucidate how the mechanism of manipulation may be a critical link to behavioural response.

## Methods

### Subjects

Sexually mature, male Mediterranean field crickets (N = 264) purchased from the local pet shop were individually housed in plastic containers (9 cm × 16 cm × 10.5 cm) covered by a plastic lid. Each container was lined with paper towels and a shelter, in the form of a cardboard tube, was provided. Crickets were held at a temperature of 23 ± 2 °C with a 12 h:12 h light:dark cycle (light on from 7 am to 7 pm) with ad libitum access to food and water (consisting of apple slices and agar water cubes). All containers were visually isolated from each other and all crickets were kept isolated for at least 12 h prior to all experiments as group living minimizes aggression in crickets^35^.

### Personality confirmation

Prior to the main study, we confirmed that the measured behaviours were repeatable in our population of crickets by behaviourally assaying 24 male crickets on three consecutive days (see description of behavioural assays below). All behaviours were repeatable (Table 1), confirming findings in other populations^26^. Thus, for our further work we only assayed individual behaviour a single time.

### Monoamine manipulation

Manipulations of both monoamine systems were based on concentrations found in the literature. Manipulation of the dopaminergic system was done using ropinirole hydrochloride (Sigma-Adrich, Sweden) diluted in phosphate-buffered saline (PBS) to a 33 µM concentration^36^. Manipulation of the serotonergic system was done using fluoxetine hydrochloride (Sigma-Aldrich, Sweden) diluted to 10 μM in PBS e.g.^15^. All experimental males (N = 144) were injected by a single experimenter (LG) and received 10 µl injected between the 4–5th segment of the abdominal cavity using a micro-syringe (Hamilton, Switzerland)^29^. Experiments were run in two blocks, a dopamine group (dopamine manipulated individuals versus control individuals) followed by a serotonin group (serotonin manipulated individuals versus control individuals). All groups had 36 individuals. All control individuals were sham injected with 10 µl PBS.

### Behavioural response

To determine if manipulated monoamine levels altered behaviour, each cricket was assayed for activity, exploration, and aggression. Trials were performed in sequence and every cricket followed the same procedure. Crickets were divided into groups of four weight-matched (±0.05 g) individuals to make aggressive dyads equivalent and to fill our four available camera setups. At time of injection, each cricket within the group was marked with a different colour combination on the pronotum so individuals could be distinguished from one another during the later aggression trial (markings used: none, red, white, red and white). Between 30 and 60 minutes post-injection, behavioural assays began^15^.

First, crickets were assessed for activity in a familiar environment (using automatic tracking with Ethovision XT 10, Noldus, 2013). Individuals in their home containers were moved to the recording setup. To optimize the automatic video tracking, the lid, shelter and food/water dishes were removed from the home container. After 10 minutes of acclimation, activity was recorded for 15 minutes as total distance moved (cm)^26^.

Immediately after the activity assay, individuals were moved in their home shelters to novel areas in the recording setup. The novel area was a larger clear plastic container (36 cm × 21.5 cm × 22 cm) with white sand. Shelters were placed in the back-left corner of the area. Exploration, defined as the total distance moved (cm) in this novel environment within 15 minutes of emergence, was measured automatically.

The final behavioural assay, aggression trials, was conducted immediately after exploration trials. The exploration areas were divided into two using an opaque cardboard divider. Individuals of the different treatments (control vs serotonin, or control vs dopamine) were placed on either side of the divider. Crickets were given 10 minutes to acclimate before the divider was raised and behaviour was observed live for 10 minutes by an observer blind to treatment^26^. We recorded the winner of each dyad as a binomial response with the first cricket to win three consecutive interactions called ‘winner’ (scored as 1), the other ‘loser’ (scored as 0). An interaction was defined as starting when any part of one cricket came in contact with any part of the other cricket and ended when that contact was aborted for more than 2 seconds^37^. An interaction was deemed as won by the cricket that produced a victory song, whilst the other cricket fled^38^. If crickets did not interact enough times for a winner to be assigned, both individuals were recorded as losers.

### Dose confirmation

We found no alteration in measured behavioural responses were explained by our dopamine manipulation (see Results). As ropinirole is not a commonly studied drug outside of humans, there is little available data on its dose-response curve, but it is likely to be non-linear^39,40^. We therefore conducted a follow up experiment to verify that we used an appropriate dose in our manipulations. We selected 6 biologically relevant dose levels ranging from the minute concentrations measured in surface waters (from human waste) to the high concentrations used for human therapeutic doses e.g.^39,41^. We again diluted ropinirole hydrochloride in PBS to obtain the specific concentrations of 0 µM (control, PBS only), 0.033 µM, 1 µM, 33 µM, 148 µM, and 330 µM. For each concentration level, 16 males were injected with 10 µl as described above (n = 96). We found that intermediately low doses (1 µM and 33 µM) tended to show a difference in behaviour between control and treated individuals, thus confirming our use of 33 µM concentrations for our main study (Table 2) and highlighting the weak effects of ropinirole on our measured behaviours.

### Statistics

All statistical analyses were conducted using the R software (version 3.4; R Development Core Team, 2017). For ‘dose confirmation’ and ‘behavioural response’ we applied linear and generalised linear mixed-effects models to analyse our data (detailed below), for which we used the ‘lmer’ and ‘glmer’ functions (package lme4)^42^. Additionally, we used the ‘sim’ function (package arm)^43^ to simulate the posterior distribution of the model parameters and values were extracted based on 2000 simulations^44^. The statistical significance of fixed effects and interactions were assessed based on the 95% credible intervals (CI) around the mean (β). We consider an effect to be “significant” when the 95% CI did not overlap zero^45^. We used visual assessment of the residuals to evaluate model fit.

#### Personality confirmation

To confirm our measured behaviours were repeatable, and thus indices of personality, repeatability calculations were calculated using the ‘rpt’ function (package rptR)^46^. Activity was log transformed to meet normality assumptions and modelled with a Gaussian distribution. Exploration (distance moved in novel area in cm) was normally distributed and modelled with a Gaussian distribution. Aggression (winner of fight) was modelled with a binomial distribution.

#### Behavioural response

We used (generalised) linear mixed models to analyse models to determine behavioural responses to our monoamine manipulations. As experiments were run independently, we ran identical but separate models to investigate the effect of manipulated levels of dopamine and serotonin. For each monoamine (dopamine, serotonin), we ran models for each response variable of interest (activity, exploration, and aggression). Activity in the serotonin manipulated group, and exploration in both dopamine and serotonin manipulated groups were non-normally distributed and so were square-root transformed. Aggression data followed a binomial distribution and was modelled as such. The models for activity and aggression were identical and included type of treatment (manipulated vs. control; categorical variable) as the fixed effect of interest. The colour marking for individual identification (none, red, white, red and white), time of injection (range: 08:30–14:00), and time since injection (30–60 min) were included as random effects. Since both exploration and activity measure the distance moved by an individual, they may be correlated^26^, thus our model of exploration included the additional fixed effect of activity score in order to model the variation in exploration alone.

#### Dose confirmation

To confirm the best dose of ropinirole, we used (generalised) linear mixed models comparing our concentration groups. We ran three models, one for each response variable of interest: activity, exploration, and aggression. Activity and exploration met normality assumptions and were modelled following a Gaussian distribution. Aggression data followed a binomial distribution and was modelled as such. All models included dose level (factor with 6 levels, one for each concentration) as the fixed effect. The colour marking for individual identification, time of injection, and time since injection were included as random effects. As described above in main study, for the model of exploration, we added the fixed effect of activity score in order to control for individual variance in activity and thus model variation in exploration alone.

## Electronic supplementary material

Supplementary Information

## Data Availability

All raw data is available in the Supplemental Information.
